# Soluble amyloid-beta buffering by plaques in Alzheimer disease dementia versus high-pathology controls

**DOI:** 10.1371/journal.pone.0200251

**Published:** 2018-07-06

**Authors:** Thomas J. Esparza, Mihika Gangolli, Nigel J. Cairns, David L. Brody

**Affiliations:** 1 Department of Neurology, Washington University, St. Louis, Missouri, United States of America; 2 Department of Biomedical Engineering, Washington University, St. Louis, Missouri, United States of America; 3 Knight Alzheimer Disease Research Center, Washington University, St. Louis, Missouri, United States of America; 4 Hope Center for Neurological Disorders, Washington University, St. Louis, Missouri, United States of America; Nathan S Kline Institute, UNITED STATES

## Abstract

An unanswered question regarding Alzheimer disease dementia (ADD) is whether amyloid-beta (Aβ) plaques sequester toxic soluble Aβ species early during pathological progression. We previously reported that the concentration of soluble Aβ aggregates from patients with mild dementia was higher than soluble Aβ aggregates from patients with modest Aβ plaque burden but no dementia. The ratio of soluble Aβ aggregate concentration to Aβ plaque area fully distinguished these groups of patients. We hypothesized that initially plaques may serve as a reservoir or sink for toxic soluble Aβ aggregates, sequestering them from other targets in the extracellular space and thereby preventing their toxicity. To initially test a generalized version of this hypothesis, we have performed binding assessments using biotinylated synthetic Aβ_1–42_ peptide. Aβ_1-42_-biotin peptide was incubated on unfixed frozen sections from non-demented high plaque pathology controls and patients with ADD. The bound peptide was measured using ELISA and confocal microscopy. We observed no quantitative difference in Aβ binding between the groups using either method. Further testing of the buffering hypothesis using various forms of synthetic and human derived soluble Aβ aggregates will be required to definitively address the role of plaque buffering as it relates to ADD.

## Introduction

The relationship between Aβ plaque pathology and Alzheimer disease dementia (ADD) [1] has been a topic of considerable controversy. Soluble Aβ aggregates have been more directly linked to toxicity *in vitro* and in some animal model systems [2]. We recently reported that soluble Aβ aggregates (termed “oligomers” in prior publications) were elevated in aqueous brain lysates from patients with early ADD in comparison with lysates from patients with Aβ plaque pathology but no dementia [3]. However, despite statistically significant differences, there was still considerable overlap between groups. In further analyses, we found that the *ratio* of soluble Aβ aggregate levels to plaque area instead fully distinguished between patients with early ADD from patients with plaques but no dementia. This finding was replicated in a second cohort of cases. While it is likely that this finding indicates a fundamentally important pathophysiological linkage underlying ADD, the interpretation of the result is not straightforward. We posited in our initial report that plaques could serve as binding reservoirs or buffers for soluble Aβ aggregates; at early stages, Aβ plaques could adequately buffer soluble aggregates, protecting the nearby neuropil from toxicity, whereas at later times if buffering capacity was lost or overwhelmed, soluble aggregates could be free to diffuse in the extracellular space and exert toxicity [3]. Direct binding and unbinding of soluble aggregates to the plaques themselves would be the most straightforward mechanism, but binding and unbinding to peri-plaque elements including dystrophic neurites, microglia, and astrocytes would be functionally equivalent. This idea was also based in part on the observations of Koffie et al., who reported loss of synapses in a gradient around plaques both in transgenic mice [4] and human brain sections [5], corresponding with a halo of immunoreactivity consistent with soluble aggregates/oligomeric species. Koffie et al. interpreted this observation as consistent with release of synaptotoxic soluble aggregates from plaques. Since synapse loss correlates strongly with dementia [6, 7] the failure of buffering leading to synaptic toxicity could be proposed as a substrate of dementia. The question of whether failure of buffering of soluble Aβ by plaques underlies ADD leads to several previously specified non-mutual exclusive hypotheses [2].

Here we report our first efforts towards testing the hypothesis that qualitative changes in Aβ plaques correlate with dementia severity; that plaques from non-demented controls retain more buffering capacity than plaques from demented patients. Specifically, we assessed Aβ buffering capacity of plaques in frozen brain slices from non-demented controls vs. patients with early dementia. While we have made initial progress in purifying soluble Aβ aggregates from human brain tissue [8], we have not yet achieved sufficient purification to use native human brain aggregates for these buffering studies. Therefore, we used biotinylated synthetic Aβ_1–42_ peptide for these initial experiments, reasoning that if plaque buffering of soluble Aβ is a general property, this approach could yield insight into the buffering properties.

## Materials and methods

### Regulatory compliance

All protocols were carried out in accordance with the Charles F. and Joanne Knight Alzheimer Disease Research Center and the Washington University Institutional Review Board, known as the Human Research Protection Office (HRPO). All donors or family members gave informed consent for a brain donation and use in research studies. The clinical characterization of research participants in life was reviewed and approved by the Washington University Human Research Protection Office (HRPO): 201105103, 201105102, 201105305. Studies involving post mortem brain tissue, including the present study, have been determined by HRPO not to constitute human subjects research since all individuals are deceased, and thus do not require separate human subjects approval. Written consent was obtained for clinical assessment of research participants in life. Capacity to consent was determined using a brief questionnaire containing the elements of consent [9]. If participants are not able to provide consent, consent is obtained from a Legally Authorized Representative (LAR). Written consent for autopsy during life, which is legal in the state of Missouri, is obtained from research participants, involving an LAR as determined to be necessary. In some instances, next-of-kin provide written consent for autopsy after death. HRPO has approved the use of an LAR to provide consent for individuals determined not to have the capacity to consent. Clinical assessments were performed and brain autopsies were obtained in individuals who in life did not have the capacity to consent. LARs provided consent in these cases. It was necessary to include individuals without the capacity to consent in this research study because the scientific question focused on Alzheimer disease dementia.

### Selection and sectioning of human frontal cortical tissue

Clinically and neuropathologicaly well-characterized cases were obtained from the Knight Alzheimer Disease Research Center, Washington University School of Medicine, Saint Louis, Missouri, USA. Brain tissue was obtained from the frontal lobe and included the cortical ribbon and underlying white matter (Brodmann areas 8/9). Cognitive status was determined during life using the Clinical Dementia Rating (CDR). At the time of expiration a final CDR was ascertained using established procedures. Alzheimer pathology was assessed using the NIA-AA neuropathologic diagnostic criteria. The samples included: CDR1 cases (n = 10, 85.2±10.8 years at death, 16.1±6.3 hours post mortem interval, 8 females & 2 males) and CDR0 with Aβ pathology cases (n = 6, 90.4±9.6 years at death, 11.7±8.5 hours post mortem interval, 4 female & 2 male).

Unfixed frontal cortex was embedded in ‘optimum cutting temperature’ (O.C.T., Sakura Finetek, USA) compound and frozen by immersion in liquid nitrogen chilled 2-methylbutane. The tissue blocks were then equilibrated at -20°C and 18μm sections were cut and mounted directly onto positively charged glass slides using a Leica CM 1950 cryostat. The tissue sections were stored at -80°C prior to use.

For quantification of Aβ plaque pathology, adjacent tissue samples were trimmed and placed into 4% paraformaldehyde for 48 hours and then transferred into 30% sucrose (w/v) in 1x phosphate buffered saline for an additional 48 hours. Free-floating 50μm sections were cut using a HM 430 sliding microtome (Thermo Fisher Scientific, Waltham, MA) and stored in a cryoprotectant solution (0.44M sucrose, 2.7M ethylene glycol, 30mM sodium phosphate buffer, pH 7.4) prior to immunohistochemistry.

### Immunohistochemical staining of human cortical tissue

Free-floating 50μm tissues sections were washed 3x in Tris-buffered saline (TBS) for 5 minutes each and then incubated with 0.3% H_2_O_2_ in TBS for 10 minutes at room temperature to block endogenous peroxidase. Following the incubation, sections were rinsed in TBS 3x for 5 minutes each, and then blocked with 5% normal goat serum (NGS) in TBS containing 0.25% (v/v) Triton X100 for 30 minutes at room temperature. Sections were then incubated with the Aβ-specific N-terminal mouse monoclonal HJ3.4 in 1% NGS in TBS-X at a 1:1000 dilution overnight at 4°C. The following day, sections were washed 3x in TBS for 5 minutes each and incubated with a biotinylated secondary goat anti-mouse antibody at a 1:1000 dilution in TBS-X for 1 hour at room temperature (Vector Laboratories). Following the incubation of the secondary antibody, the sections were washed 3x in TBS for 5 minutes each, incubated with ABC Elite (Vector Laboratories) at a 1:400 dilution in TBS for 1 hour at room temperature, then washed with TBS 3x for 5 minutes and developed with 3,3’-diaminobenzidine (#D5905; Sigma-Aldrich). Sections were mounted and dehydrated using a standard ethanol-xylene series followed by coverslipping. For each patient, three tissue sections separated by 600μm were stained for analysis.

### Quantification of immunohistochemical staining with ImageJ

Immunohistochemical samples from each patient were scanned using a Hamamatsu NanoZoomer HR model (Hamamatsu, Bridgewater, NJ). The percentage of gray matter containing HJ3.4 immunopositive Aβ plaque was measured using the Analyze Particles ImageJ (NIH) plug-in on thresholded 8-bit images with user defined gray-matter distinct regions of interest. The image thresholding was performed equally across the full sample set. During quantitation, the samples were coded such that the user was masked to the patient ID and CDR status.

### Binding of Aβ_1-42_-biotin to unfixed frozen human cortical tissue

For the binding experiments, unfixed frozen tissue sections (n = 6 per patient) were equilibrated to ambient temperature. The residual O.C.T. compound was gently removed from surrounding the tissue and a hydrophobic barrier surrounding the tissue was prepared using an ImmEdge PAP pen (H-4000, Vector Laboratories, Burlingame, CA). Non-specific blocking of the tissue was performed using 200μl of a 1% bovine serum albumin (A7030, Sigma-Aldrich, St. Louis, MO) in artificial cerebral spinal fluid (aCSF) solution (148mM sodium chloride, 3mM potassium chloride, 1.4mM calcium chloride, 0.8mM magnesium chloride, 0.8mM sodium phosphate dibasic, 0.2mM sodium phosphate monobasic) for 1 hour at room temperature in a humidified chamber. The blocking solution was replaced with 150μl binding buffer (0.05% BSA in aCSF) containing 10nM N-terminal biotinylated synthetic Aβ_1–42_ (AS-23523, Anaspec, Fremont, CA) and incubated for 18 hours at room temperature in a humidified chamber. Following overnight binding, the tissue sections were gently washed 3x for 5 minutes each with binding buffer at room temperature. For direct quantification of the bound biointylated Aβ_1–42_ the tissue was dissociated using concentrated formic acid (n = 3 sections/patient) and aliquoted into sterile microcentrifuge tubes. The formic acid in each sample aliquot was removed by vacuum centrifugation and the samples stored at -80°C until assayed. For confocal microscopy, the remaining sections (n = 3/patient) were fixed with 4% paraformaldehyde in chilled methanol. The N-terminal biotinylated synthetic Aβ_1–42_ was prepared by incubation with hexafluoroisopropanol (HFIP) overnight without sonication and then the solvent removed by evaporation at room-temperature under nitrogen atmosphere. The resulting film was resuspended in DMSO and stored at -80°C in single-use aliquots. A single batch of peptide was used for the entire set of experiments.

### Assessment of *ex-vivo* binding of Aβ_1-42_-biotin by confocal microscopy

The Aβ_1-42_-biotin bound tissue sections (n = 3/patient) were subjected to signal amplification to allow for confocal image acquisition. The fixed sections were washed 3x in aCSF for 5 minutes each and incubated with ABC Elite at a 1:400 dilution in aCSF for 1 hour at room temperature. The signal was then amplified using the Biotin-XX Tyramide SuperBoost Kit (B40931, Thermo Fisher Scientific) with a development time of 10 minutes followed by washing 3x in aCSF for 5 minutes each. To quench endogenous autofluorescence the tissue sections were treated with TrueBlack (23007, Biotium, Fremont, CA) for 2 minutes followed by extensive washing in aCSF to remove residual reagent. The quenched, amplified sections were then incubated with streptavidin Alexa Fluor™ 594 conjugate (S11227, Thermo Fisher Scientific) at 1:1000 diluted in 0.1% BSA in aCSF for 30 minutes. The sections were washed 3x in aCSF for 5 minutes each, followed by counterstain with 4′,6-diamidino-2-phenylindole (DAPI, Sigma-Aldrich), and a final wash series before mounting with ProLong Gold Antifade media (P10144, Thermo Fisher Scientific). Images were acquired using an Olympus FV1200 scanning confocal microscope equipped with gallium arsenide phosphide detectors. Tiled (4x5), 7.5 micron z-stack images were acquired using a UPLFLN 20x (NA:0.70) objective with a laser output (0.5%) that produced non-saturated pixel data. All patient sections were acquired using the same instrument settings.

### Quantification of confocal imaging with ImageJ

Confocal acquired tiled images files (Olympus .OIB format) from each patient were imported into ImageJ for analysis. Using the DAPI channel, a global region of interest was defined to exclude non-tissue area. The Alexa Fluor™ 594 channel was converted to a z-projection using the “sum of slices” setting. A duplication of the z-projection was converted to an 8-bit image and used to create a threshold overlay mask within the Analyze Particles plugin. The overlay masks were imported into the ROI manager. The original z-projection was converted to an 8-bit image and the global ROI was used to deselect any overlay mask outliers. The global ROI and the overlay masks were used to measure the integrated density and area measures for each image. An additional measurement was made in the non- Alexa Fluor™ 594 region to serve as a background subtraction control. The image thresholding was performed equally across the full sample set. During quantitation, the samples were coded such that the user was masked to the patient ID and CDR status.

### Measurement of Aβ_1–42_ biotin by ELISA

Measurement of the formic acid soluble recovered N-terminally biotinylated Aβ_1–42_ was determined by ELISA using the mid-domain binding antibody HJ5.1 to capture and poly-streptavidin HRP-20 to detect bound peptide. HJ5.1 was coated to 96-well Nunc Maxisorp plates at 20 μ g/mL in carbonate buffer (35 mM sodium bicarbonate, 16 mM sodium carbonate, 3 mM sodium azide, pH 9.6) in 100 μl/well overnight at 4°C. Plates were washed 5x between steps with PBS using a BioTek EXL405 plate washer. The assay plates were blocked using 0.2 μm filtered 4% BSA in PBS for 1 hour at room temperature. Samples and standard were diluted in standard diluent, as described above, to a 100μL volume and loaded. An 8-point standard curve was generated using 5000, 1666.7, 555.6, 185.2, 61.7, 20.6, and 6.9 pg/mL of the N-terminal biotinylated synthetic Aβ_1–42_ loaded in triplicate. All samples were kept on ice during handling and the assay plates were incubated at 4°C overnight prior to development.

### Measurement of total Aβ _1-x_ by ELISA

The quantification of the Aβ_1-x_ isoforms was performed as previously described [8]. The samples were resuspended in 5M guanidine-HCl prior to dilution with standard buffer (0.2 μm filtered 0.25% BSA, 0.5 M guanidine-HCl, 0.005% Tween-20, 300 mM Tris, 3 mM sodium azide, 2 μg/mL aprotinin, 1 μ g/mL leupeptin, in PBS). The samples were loaded onto HJ5.1 coated 96-well Nunc Maxisorp plates, which were blocked with 4% BSA, in addition to synthetic Aβ_1–40_ monomer standard loaded in triplicate. Following overnight incubation, the assay plates were detected using biotinylated HJ3.4 which binds N-terminally intact Aβ but should not bind to N-terminally biotinylated Aβ_1–42_. The assay was developed as previously described using poly-streptavidin HRP-20 (65R-S103PHRP, Fitzgerald, Acton, MA) and addition of 3,3’,5,5’-tetramethylbenzidine (T5569, Sigma-Aldrich) with the absorbance read on a BioTek Synergy 2 plate reader at 650 nm.

### Total protein quantification

Total protein was quantified in the formic acid dissociated samples using a fluorescence-based 96-well NanoOrange assay (N6666, Thermo Fisher Scientific). Sample aliquots were resuspended in 4M urea as well as dilutions of the reference BSA standard to normalize for effect on the assay. Appropriate dilutions for each samples and the standard were combined with assay reagent in a 96-well black microplate and measured by excitation at 485nm and emission at 590nm using a BioTek Synergy 2 plate reader.

### Assessment of Aβ_1-42_-biotin during binding by size-exclusion chromatography

To determine if qualitative size changes or quantitative loss of Aβ_1-42_-biotin occurred during the room-temperature overnight incubation period, we assessed pre- and post-incubation samples by size-exclusion chromatography. Clean slides without tissue were prepared and blocked the same as for the tissue sections. The blocking solution was replaced with 150μl binding buffer (0.05% BSA in aCSF) containing 10nM N-terminal biotinylated synthetic Aβ_1–42_ and incubated for 18 hours at room temperature in a humidified chamber. For the pre-incubation sample, the peptide solution was immediately recovered into a blocked-microcentrifuge tube. The recovered sample was injected onto a pre-equilibrated Superdex 200 10/300 GL column pre-equilibrated with 0.05% BSA in aCSF. The sample was eluted at 0.5 ml/min at 4°C and then immediately diluted and loaded onto an ELISA plate coated with HJ5.1 for quantitative analysis. The overnight incubation sample was recovered and assessed by size-exclusion chromatography in the same manner.

### Statistical methods

All data were analyzed using Prism 7.0 (GraphPad Software, La Jolla, CA). The Shapiro-Wilk normality test was used to determine whether the measures were normally distributed. In each assessment at least one group was observed to be non-normally distributed, therefore, the non-parametric Mann-Whitney U test was used.

## Results

### Plaque coverage and morphology was indistinguishable between non-demented controls vs. patients with early ADD

**Fig 1:**
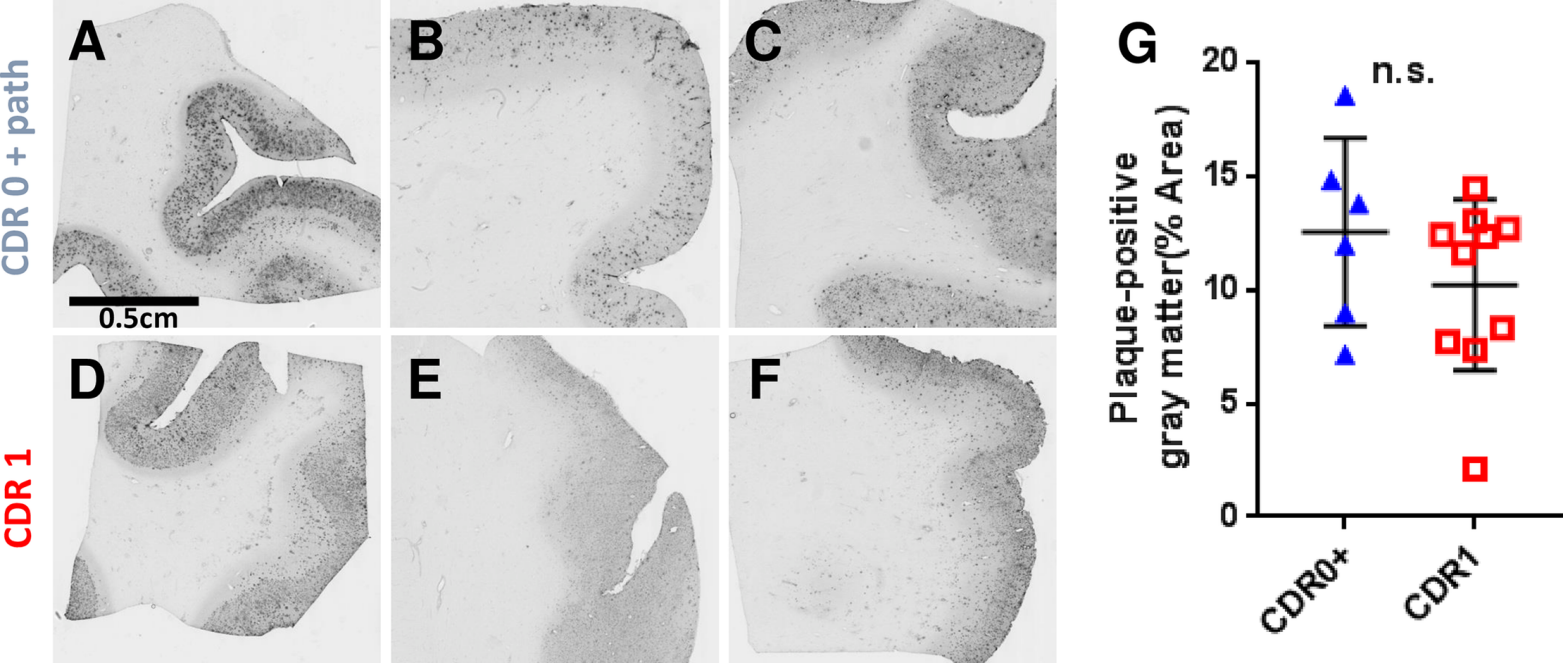
Representative amyloid-beta (Aβ) immunohistochemistry demonstrates similar levels of pathology between elderly high-pathology controls and elderly subjects with mild ADD. Scale bar = 0.5 cm, applies to panels A-F. (**A-C**) Aβ plaque pathology in frontal cortex sections from nondemented elderly subjects (CDR 0 + plaques). (**D-F**) Aβ plaque pathology in frontal cortex sections from elderly subjects with mild ADD (CDR 1). (**G**) Gray matter coverage by Aβ plaque pathology was not different in the nondemented elderly subjects with plaques (CDR 0 + plaques) versus subjects with mild ADD (CDR 1) (not significant [n.s.] by Mann–Whitney U test).

As demonstrated in our previous report[3], Aβ plaques immunoreactive with the Aβ N-terminal specific antibody HJ3.4 covered similar portions of the gray matter in non-demented controls compared to patients with early ADD (**Fig 1**). The brain samples used in these experiments came from different cases than those used for our previous report, but the demographics of the patients and the characteristics of the Aβ plaque pathologies were very similar (**Table 1**). In addition, there are no significant differences in post-mortem interval (PMI) or the age at death between groups.

**Table 1:** Characteristics of human brain frontal cortex samples.

Patient No.	Status	Age (yr.)	PMI (hr.)	Gender
1	CDR 0 + path	97	3.5	M
2	CDR 0 + path	91.7	12	F
3	CDR 0 + path	100.9	21	F
4	CDR 0 + path	95.4	23	F
5	CDR 0 + path	80.8	5.5	M
6	CDR 0 + path	76.7	5	F
7	CDR 1	86.4	6.7	F
8	CDR 1	89	19	F
9	CDR 1	92.7	23	F
10	CDR 1	94.2	11.6	F
11	CDR 1	104.4	19	F
12	CDR 1	68.6	21	M
13	CDR 1	86	18	F
14	CDR 1	81	21	F
15	CDR 1	72.7	4.5	M
16	CDR 1	76.8	17	F

### No difference between binding of biotinylated Aβ_1–42_ bound to plaques in non-demented controls vs. plaques in patients with early ADD: Results from confocal imaging

**Fig 2:**
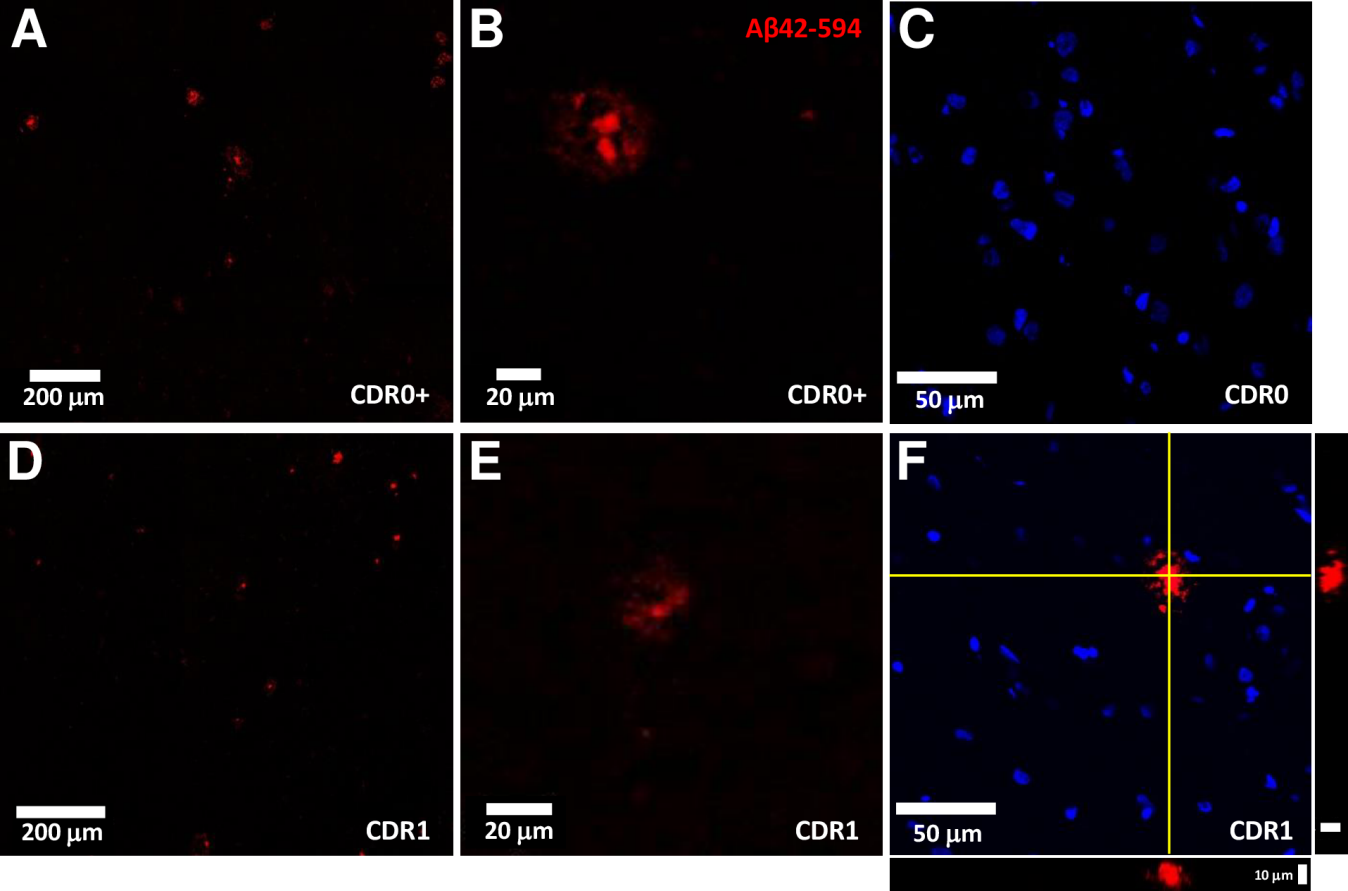
Exemplar fluorescent confocal microscopy of Aβ_1-42_-biotin binding to unfixed, frozen frontal cortex sections. (**A, D**) Representative fluorescent confocal images of labelled (streptavidin-Alexa594, red channel) Aβ_1-42_-biotin binding in elderly high-pathology controls (CDR0 + plaques) and elderly subjects with mild ADD (CDR1). Scale bar = 200 μm. (**B, E**) Higher magnification confocal images reveal distinct plaque morphology and minimal background fluorescence in both in elderly high-pathology controls (CDR0 + plaques) and elderly subjects with mild ADD (CDR1). Scale bar = 20 μm. **(C)** Fluorescent images of labelled (streptavidin-Alexa594, red channel) Aβ_1-42_-biotin binding display an absence of plaque structure morphology with minimal background signal in cognitively normal elderly subjects without plaque pathology. Nuclei stained with DAPI (blue channel). Scale bar = 50 μm. **(F)** Fluorescent images of labelled (streptavidin-Alexa594, red channel) Aβ_1-42_-biotin binding display punctate staining of a central core with peripheral decoration in elderly subjects with mild ADD (CDR1). Nuclei stained with DAPI (blue channel). Scale bar = 50 μm. Orthogonal XZ and YZ views, centered on the yellow crosshairs, demonstrate the labelling extent through the tissue section. Scale bar = 10 μm.

**Fig 3:**
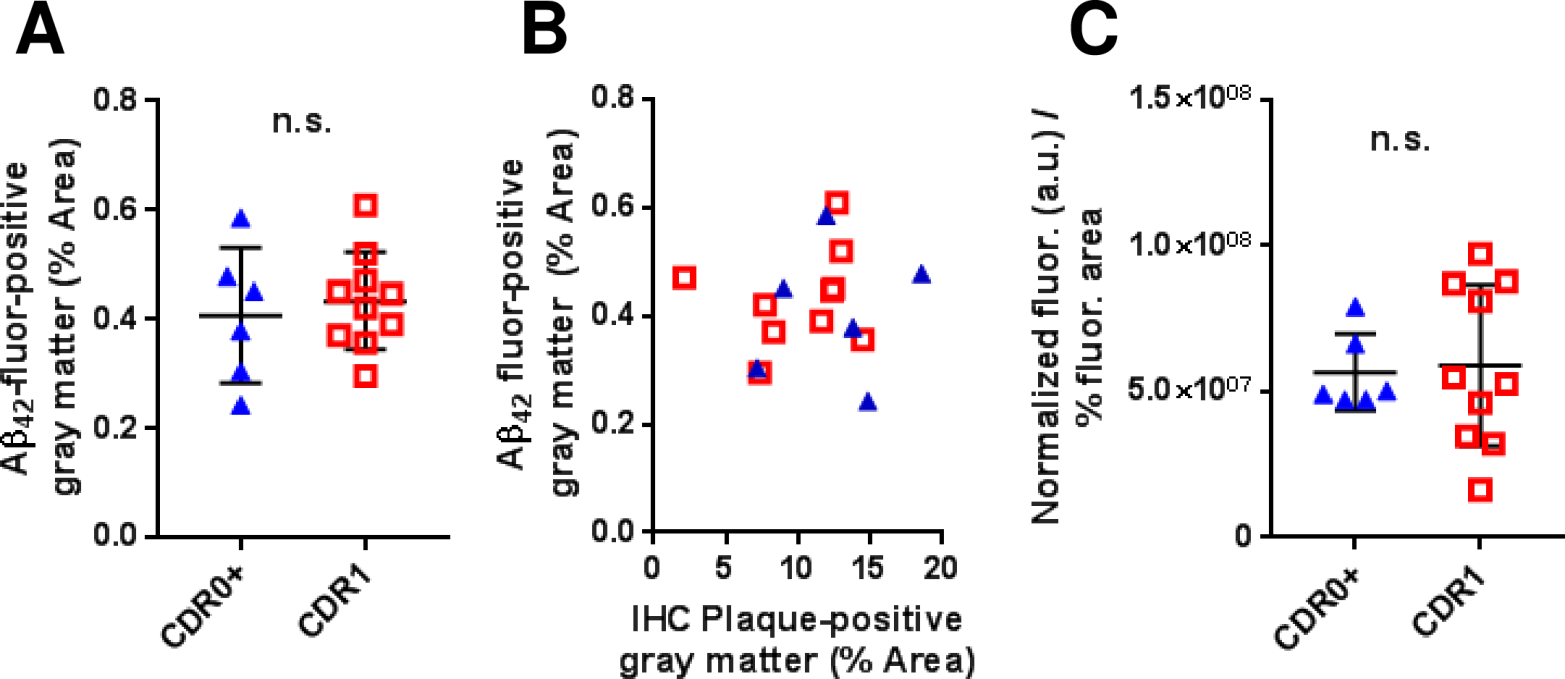
Assessments based on confocal fluorescence microscopy of Aβ_1-42_-biotin binding does not distinguish between elderly high-pathology controls and elderly subjects with mild ADD. (**A**) Gray matter coverage of fluorescently labelled (streptavidin-Alexa594) Aβ_1-42_-biotin binding in CDR 0 + plaques group versus CDR 1 group (not significant [n.s.] by Mann–Whitney U test). (**B**) Correlations between fluorescently labelled Aβ_1-42_-biotin gray matter coverage and overall Aβ plaque coverage. (**C**) Ratio of the normalized fluorescently labelled Aβ_1-42_-biotin signal to the percentage fluorescent positive coverage (not significant [n.s.] by Mann–Whitney U test).

After binding of soluble biotinylated Aβ_1–42_ for 18 hours at room temperature to frozen sections followed by gentle washing in binding buffer (0.05% BSA in artificial CSF), biotinylated Aβ_1–42_ was retained in structures similar in size and morphology to Aβ plaques and did not appear to non-specifically bind throughout the tissue (**Fig 2A, 2B and 2D–2F**). No binding was detected in brain sections from patients without plaques (**Fig 2C**), or on blank slides. Binding of biotinylated Aβ_1–42_ covered 0.407+/-0.124% of the brain area in slices from non-demented controls and 0.433+/-0.089% of the area in slices from patients with early dementia. The area covered did not differ between groups (**Fig 3A**¸ p = 0.6299). The area covered by bound soluble biotinylated Aβ_1–42_ did not correlate with the total immunohistochemically detected plaque coverage (**Fig 3B**). The total fluorescence intensity also did not differ between groups (p = 0.9463). After normalization by total Aβ plaque area, total fluorescence intensity similarly did not differ between groups (**Fig 3C**). Thus, the binding capacity of plaques for synthetic biotinylated Aβ_1–42_ does not appear to differentiate demented from non-demented high plaque control brains.

### No difference between binding of biotinylated Aβ_1–42_ bound to slices from non-demented controls vs. slices from patients with early ADD: Results from ELISA

**Fig 4:**
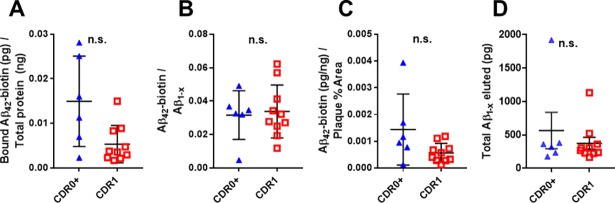
Assessments based on enzyme-linked immunosorbent assay of bound Aβ_1-42_-biotin does not distinguish between elderly high-pathology controls and elderly subjects with mild ADD. (**A**) There was no difference between groups in the levels of overall Aβ_1-42_-biotin recovered from dissociated tissue, as measured using an indirect ELISA which only detects biotinylated Aβ (not significant [n.s.] by Mann–Whitney U test). Data expressed as picograms Aβ per nanogram of total measurable protein. (**B**) The ratio of the amount of Aβ_1-42_-biotin to the amount of Aβ_1-x_ as measured by sandwich ELISA was not different between groups (not significant [n.s.] by Mann–Whitney U test). (**C**) The ratio of Aβ Aβ_1-42_-biotin as measured by sandwich ELISA to the percent gray matter plaque coverage did not differ between groups (not significant [n.s.] by Mann–Whitney U test). (D) There was no difference between groups in the levels of overall Aβ_1-x_ recovered from dissociated tissue, as measured using an sandwich ELISA (not significant [n.s.] by Mann–Whitney U test).

Confocal imaging is most sensitive to tightly localized fluorescence, such as that due to binding of Aβ to discrete plaques and peri-plaque structures. However, it is also possible that other brain tissues could bind and buffer Aβ more diffusely, which might be difficult to detect using confocal microscopy. Therefore, we also used a parallel biochemical approach in which we incubated separate sets of frozen brain slices from the same patients with biotinylated Aβ_1–42_, washed, and then lysed the slices in formic acid to solubilize all of the Aβ in the slices for measurement by ELISA. In agreement with the previous results, we found no difference between the biotinylated Aβ_1–42_ in the lysates from slices from non-demented controls vs. slices from patients with early ADD (**Fig 4A**, p = 0.0559). There were no differences between groups after normalizing by total Aβ_1-x_ in the lysates (**Fig 4B**, p = 0.7925), nor after normalizing by Aβ plaque coverage (**Fig 4C**, p = 0.0934). There were no differences between groups in the level of total Aβ_1-x_ measured from tissue elution (**Fig 4D**, p = 0.4923). Thus, an orthogonal approach to measurement of buffering capacity similarly did not differentiate demented from non-demented patients.

### Synthetic Aβ_1–42_ is partially aggregated at baseline and aggregates further over time during the binding experiments

**Fig 5:**
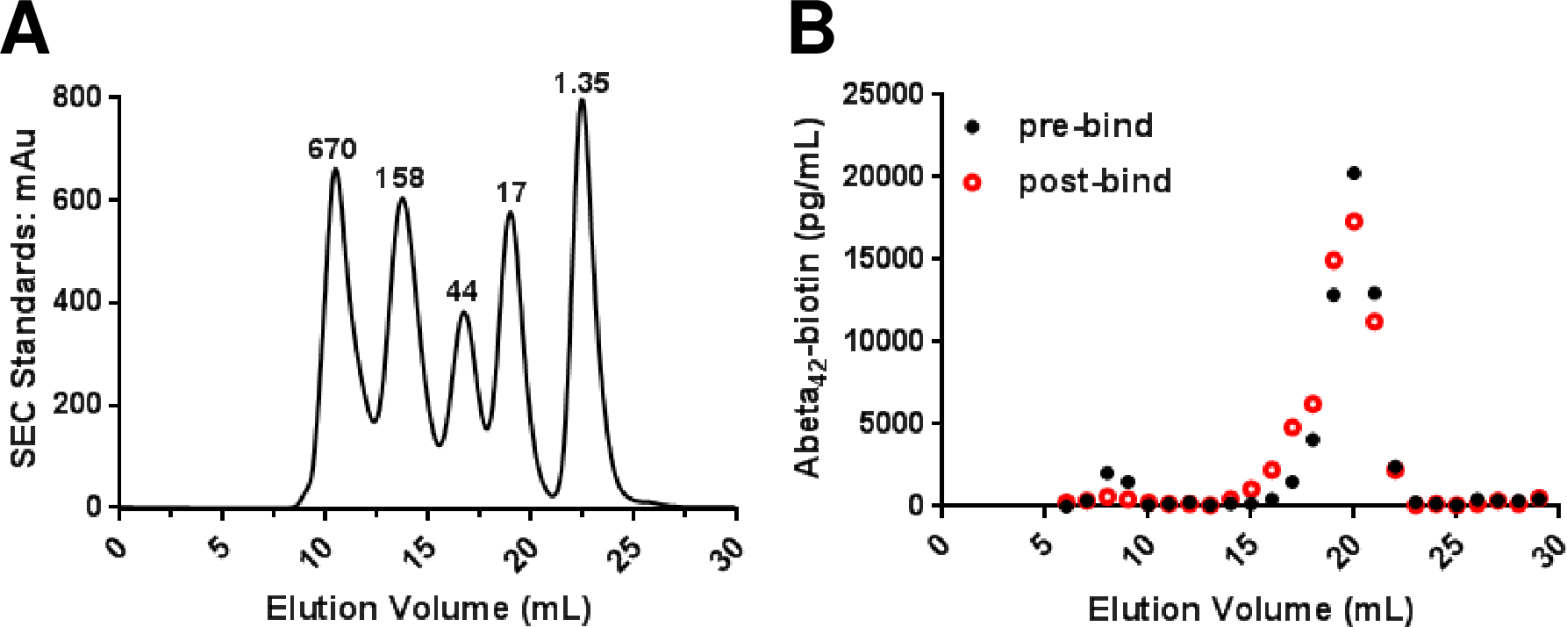
Size exclusion chromatography reveals that the Aβ_1-42_-biotin monomer remained predominantly low molecular weight during the experiment. (**A**) Globular protein molecular weight standards as run on a size exclusion Superdex 200 10/300 GL column. mAu = milli-absorbance units, kDa = kilodaltons. (**B**) The predominance of the pre-incubation (black closed circles) Aβ_1-42_-biotin elutes as a low molecular weight peak in fractions 18 to 21 likely corresponding to monomer, and a minority of the Aβ_1-42_-biotin eluted as a high molecular weight peak in fractions 8 and 9 corresponding to aggregated Aβ. The post overnight incubation sample (red open circles) has a minor shift to include a shoulder of fractions 16 and 17 in the low molecular weight peak suggesting the accumulation of oligomeric species while most the sample remained monomeric.

Synthetic Aβ_1–42_ can be induced to form a large number of aggregation forms, from dimers to higher order oligomers. We performed size exclusion chromatography (SEC) under neutral pH conditions to examine the size forms of the synthetic Aβ_1–42_ in the solutions that were applied to the slices, and again to examine the size forms present in the supernatant from the slices after the 18 hour incubation on slices at room temperature. At baseline, most of the Aβ_1–42_ appeared monomeric, running in fractions 18 to 22 between the 17kDa and 1.35kDa size standards (**Fig 5B**–black solid circles). A small portion appeared to be high molecular weight running in fractions 8 to 9 equivalent to the 670kDa size standards. The pre-incubation SEC fractions 8 to 9 (1787pg/mL) demonstrated a reduction in quantity following incubation (533pg/mL). After incubation, the Aβ was still mainly monomeric, but a portion had shifted to slightly larger appearing forms, in fractions 16 to 18 between the 44kDa and 17kDa size standards (**Fig 5B**–red open circles). The average quantity of Aβ_1–42_ in the pre-incubation and post-incubation SEC fractions 18 to 22 (12,546pg/mL and 12445pg/mL respectively) remained quite consistent during the slight increase in size distribution following incubation. This contrasts with almost twice the amount of Aβ_1–42_ in the post-incubation SEC fractions 16 to 18 (4429pg/mL) compared with the equivalent pre-incubation fractions (2011pg/mL).

## Discussion

In summary, synthetic biotinylated Aβ_1–42_ binds to plaque-like structures indistinguishably in frozen brain slices from demented vs. non-demented high Aβ plaque pathology donors. This negative result does not support the hypothesis that a generalizable differential buffering of Aβ by plaques underlies the difference between demented and non-demented high plaque pathology patients. However, appropriate methods to definitively address the plaque buffering hypothesis have not yet been developed. Most importantly, native soluble Aβ aggregates may have very different properties from synthetic biotinylated Aβ_1–42_ due to differences in proteoforms [10], aggregation states, and potentially associated proteins [2].

### Relationship to previous studies

From a methodological perspective, our approach has similarities to those used previously by others. For example, Guo et al. [11] demonstrated that exogenously applied fluorescently labelled Aβ_1–42_ bound plaques in unfixed frozen human cortical sections, though their binding assays were performed for 30 minutes at 37°C rather than over 18 hours at room temperature as in our studies. Esler et al. [12] reported that synthetic Aβ exposed to human cortical sections for a short time unbound quickly upon washing, whereas synthetic Aβ exposed for longer times unbound at substantially slower rate. These results, and others, led to the two process ‘dock-lock’ model of soluble Aβ interacting with plaques. We specifically used an 18 hour binding time to attempt to assess both processes in our studies. Tseng et al. [13] reported that while monomeric Aβ_1–40_ bound plaques from AD brain, aged high molecular weight synthetic Aβ aggregates did not show detectible plaque binding. Concordantly, the majority of the Aβ in our experiments was monomeric throughout the experiment, though there appeared to be some aggregation over time. However, none of these previous investigations compared Aβ binding to slices from brains of patients with dementia vs. from patients with indistinguishable plaque burden but no dementia.

The above discussed studies involved binding to *ex vivo* human brain tissue plaques, whereas other groups have investigated similar properties in living animal models. For example, Gureviciene et al. [14] infused fluorescently labelled monomeric Aβ into APPswe/PSEN1dE9 mice and measured incorporation of the Aβ by two-photon excitation fluorescence microscopy. They reported accumulation of fluorescent Aβ preferentially around plaques compared to other brain regions. This result was interpreted as consistent with plaque buffering of soluble Aβ *in vivo*.

### Limitations and future directions

The main limitation of our approach was that we used synthetic monomeric Aβ. The presence of the N-terminal biotin modification in the peptide has the potential to alter some aspect of the binding affinity of the peptide to the plaques. A potential remedy could be the use of stable isotope labelled synthetic peptide followed by mass spectrometric quantification [15] to distinguish exogenous from endogenous peptide. Other groups have prepared stable high-molecular weight assemblies, such as amyloid-beta derived diffusible ligands (ADDLs)[16–18], which could provide direct comparison between higher molecular weight assemblies and the predominantly monomeric Aβ in our study. Future studies will be required to assess the properties of human brain-derived Aβ monomers and soluble Aβ aggregates. Importantly, the characteristics of native human brain-derived Aβ may not be comparable to those of synthetic Aβ aggregates due to the wide variety of proteoforms, aggregation states, and potentially co-associated proteins. For example, Jin et al.[19] demonstrated that synthetic Aβ dimers induced tau hyperphosphylation in cultured neurons at concentrations orders of magnitude less than human brain-derived Aβ immunoreactive species of comparable apparent size suggesting unique properties in the human brain-derived samples. Similarly, Noguchi et al. [20] reported that native human brain high molecular mass Aβ aggregates were substantially more toxic than similar size synthetic Aβ aggregates.

The ability to label Aβ aggregates extracted and purified from the human brain without disrupting their native tertiary structure will be essential for these future endeavors. Having methods to compare the initial state and the labelled state will be important to ensure that experiments can be interpreted accurately. We envision testing for whether the process of labeling native brain Aβ aggregates disrupts their properties by using a competition assay; for example if the directly measured affinity of labeled native brain Aβ is similar to the inferred affinity of unlabeled native brain Aβ as a competitor, this would be reassuring.

Ongoing work in our group and that of others should soon begin to address the hypothesis that qualitative differences exist in the soluble Aβ aggregates. This hypothesis will be tested by directly determining the Aβ proteoform composition of soluble Aβ aggregates extracted from the brains of patients with dementia vs. patients with indistinguishable plaque burden but no dementia. From there, the ability of plaques to buffer soluble Aβ aggregates from the two patient sources or synthetic versions of differentially abundant proteoforms will be tested directly.

A second limitation involves the use of frozen *ex vivo* brain slices. If active cellular processes play a role in the functional buffering of Aβ, this would not be detectible in our assays. In theory, microdose PET tracer labeled Aβ could be infused into local regions in the brains of patients undergoing other clinically indicated neurosurgical procedures such as shunting for normal pressure hydrocephalus. Retention vs. clearance of the PET tracer label could be then compared between non-demented low plaque, non-demented high plaque, and demented high plaque individuals.

A third limitation is that we did not measure the full kinetic binding properties of the Aβ in our experiments, nor did we measure binding at physiological temperature. It is possible that differences between the two groups in the kinetics of Aβ binding could be important, even though the likely near-equilibrium binding we measured did not differ between groups.

### Implications

A more complete understanding of the differences between the Aβ found in the brains of patients with dementia vs. patients with equivalent presence of plaque burden but no dementia may be key to developing Aβ-targeted therapeutics for ADD. It is quite possible that despite many years and many clinical trials, the appropriate Aβ therapeutic targets most relevant to dementia still have not been identified.
